# Will Teacher-AI Collaboration Enhance Teaching Engagement?

**DOI:** 10.3390/bs15070866

**Published:** 2025-06-26

**Authors:** Lai-Jian Ding, Jia-Min Li, Bei-He Hui

**Affiliations:** 1 Department of Physical Education, Zhejiang Sci-Tech University, Hangzhou 310018, China; dinglaijian@zstu.edu.cn; 2 School of Business Administration, Northeastern University, Shenyang 110819, China; 2210498@stu.neu.edu.cn

**Keywords:** teacher-AI collaboration, teaching engagement, technological self-efficacy, perceived organizational support, conservation of resources theory

## Abstract

Against the backdrop of the widespread integration of Artificial Intelligence (AI) into educational practices, collaboration between teachers and AI is profoundly influencing teaching behavior. Drawing on the Conservation of Resources Theory, this study constructs and tests a model examining the impact of teacher-AI collaboration on teaching engagement, with a focus on the mediating role of technological self-efficacy and the moderating role of perceived organizational support. Based on empirical data collected through a survey in China, the results reveal that teacher-AI collaboration significantly and positively predicts teaching engagement. Furthermore, technological self-efficacy mediates this relationship, suggesting that AI collaboration enhances teaching engagement by boosting teachers’ confidence in using technology. In addition, perceived organizational support positively moderates the effect of teacher-AI collaboration on technological self-efficacy, forming a moderated mediation model. This research enriches the understanding of teacher behavior in the context of AI integration and offers practical implications for educational institutions seeking to optimize AI adoption strategies and enhance teacher motivation.

## 1. Introduction

In recent years, the widespread application of artificial intelligence (AI) technologies in the field of education has been reshaping the teaching ecosystem at an unprecedented pace (Li et al., 2023; Michel-Villarreal et al., 2023). AI has made significant breakthroughs in areas such as intelligent recommendation, automated grading, and learning analytics, and is increasingly penetrating core aspects of teaching, including instructional design, classroom management, and student assessment (Crompton & Burke, 2023). This trend is accelerating a shift from traditional human-centered teaching models toward “human–AI collaboration”. In the emerging literature, this shift is often framed as the development of “co-intelligence”, a synergistic configuration in which human pedagogical expertise and algorithmic computation jointly create superior instructional capacity (Wilson & Daugherty, 2018). In this transformation, the role of teachers is no longer limited to knowledge transmission; instead, teachers are becoming collaborators with AI systems, jointly undertaking teaching tasks with intelligent algorithms (Neumann et al., 2023; Sun et al., 2025). From a broader behavioral-science standpoint, teacher-AI interaction constitutes a theoretically rich testbed for examining how humans allocate effort, adapt roles, and negotiate autonomy when working alongside intelligent agents—processes that also shape AI adoption in healthcare, finance, and other knowledge-intensive sectors. Viewed through the lens of Conservation of Resources (COR) theory (Hobfoll & Shirom, 2000), such collaboration can trigger either a resource-gain spiral—by freeing time and cognitive energy—or a resource-loss spiral when the technological demands outweigh the benefits. Indeed, recent studies report that configuring and supervising AI tools may lengthen lesson preparation, heighten cognitive load, and diminish perceived autonomy, thereby increasing rather than decreasing teachers’ workload (Bates et al., 2020; Wang et al., 2021). Therefore, teacher-AI collaboration is not only a practical manifestation of technological integration but also a key driver of the intelligent transformation of education (Aler Tubella et al., 2024; Wu et al., 2025). By positioning teachers as prototypical knowledge workers and mapping their resource dynamics under AI augmentation, the present study offers behavioral-science insights that generalize beyond schooling—illustrating how co-intelligence can amplify or erode engagement in any domain where human expertise and algorithmic power intersect. Recognizing both its potential gains and hidden costs is thus essential for understanding how teachers establish effective collaboration with AI systems. Understanding how teachers establish effective collaborations with AI and how such collaborations influence their teaching behavior has become a crucial direction in educational technology research.

Under the context of teacher-AI collaboration, the nature and format of teaching tasks have undergone significant changes, directly influencing teachers’ attitudes and behaviors toward teaching (Li et al., 2025a; Wang et al., 2021). Specifically, AI can handle repetitive and standardized tasks such as grading and attendance tracking, thereby freeing teachers’ time and energy for higher-order instructional activities and personalized student support (Hannan & Liu, 2023; Meng et al., 2025). From the perspective of Conservation of Resources Theory (Hobfoll & Shirom, 2000), these time and energy savings constitute valuable resources that should enhance teaching engagement; yet, COR also cautions that if new technologies consume more resources than they generate, engagement will decline. Empirical findings echo this warning: to integrate and supervise AI tools, teachers often invest additional hours in prompt design, data verification, and troubleshooting—efforts that may increase rather than decrease their overall workload (Bates et al., 2020; Wang et al., 2021). However, the integration of technology may also bring uncertainty and adaptation pressures, such as a perceived loss of instructional autonomy or increased dependence on technology, which may affect teachers’ engagement in the teaching process (Bates et al., 2020). Teaching engagement refers to the level of cognitive, emotional, and behavioral investment teachers demonstrate in their teaching activities. It is a key indicator that directly affects teaching quality and student learning outcomes (Klassen et al., 2012). Previous studies have shown that high levels of teaching engagement can enhance instructional innovation and job satisfaction while reducing teacher burnout and attrition (Klassen et al., 2012). Therefore, investigating how teacher-AI collaboration influences teaching engagement holds both theoretical and practical significance.

To gain deeper insights into the mechanisms underlying the effect of teacher-AI collaboration on teaching engagement, this study introduces technological self-efficacy as a mediating variable, grounded in the Conservation of Resources Theory. According to this theory, individuals are motivated to protect existing resources and acquire new ones when facing environmental stressors (Hobfoll & Shirom, 2000). In the context of technological transformation, the widespread use of AI constitutes a form of “technological stressor”. Teachers may experience pressure to update their knowledge, uncertainty about their redefined roles, and anxiety over potential technological failures (Huffman et al., 2013; Li et al., 2024). In this process, technological self-efficacy—teachers’ perceived confidence and competence in using AI tools for teaching—emerges as a critical psychological resource (Holden & Rada, 2011). Teachers with high technological self-efficacy are more likely to actively adapt to the AI environment and explore the value of AI tools in teaching, thereby enhancing their teaching engagement (Rahman et al., 2016). Conversely, low technological self-efficacy may lead to avoidance of technology and decreased participation (Li et al., 2025b; Pan, 2020). Hence, whether teacher-AI collaboration can effectively enhance teaching engagement largely depends on whether teachers possess sufficient confidence and perceived capability in using technology.

While technological self-efficacy plays a vital role in how teachers adapt to AI-based teaching, the formation and transformation of individual resources are often influenced by the external environment. According to the Conservation of Resources Theory, external resources—particularly organizational-level support—can mitigate the risks associated with resource depletion during technological change and enhance individuals’ capacity to cope with stress (Hobfoll & Shirom, 2000). Therefore, this study further introduces perceived organizational support as a moderating variable to examine its role in the relationship between teacher-AI collaboration and technological self-efficacy. Perceived organizational support refers to teachers’ subjective perceptions of the support provided by schools or educational administrators in terms of technical training, emotional encouragement, and institutional safeguards (Nayir, 2012). When teachers perceive strong organizational support, they are more likely to develop a sense of security and belonging, which can boost their confidence in facing technological changes and stimulate their motivation to engage in technology learning (Demir, 2015). In other words, under high levels of organizational support, teachers are more likely to gain positive experiences from AI collaboration and strengthen their technological self-efficacy (Demir, 2015). Conversely, in the absence of adequate institutional and emotional support, teachers may experience greater technological anxiety, resulting in diminished technological self-efficacy (Holsblat, 2014). Thus, perceived organizational support plays a crucial moderating role in helping teachers adapt to AI environments and build their resource base.

In summary, this study constructs a moderated mediation model based on the Conservation of Resources Theory to explore how teacher-AI collaboration influences teachers’ teaching engagement through technological self-efficacy, and further examines the moderating role of perceived organizational support in this mechanism. This research addresses a theoretical gap in understanding teacher behavior amid the ongoing intelligent transformation of education and provides both theoretical and practical implications for enhancing teachers’ adaptability and improving the implementation of AI technologies in educational settings. By uncovering the mechanisms through which individual psychological resources and organizational support resources function during technological transitions, this study aims to offer valuable insights for future teacher professional development, instructional design innovation, and educational policy-making.

## 2. Theoretical Framework and Hypothesis Development

### 2.1. Theoretical Foundation: Conservation of ResourcesTheory

Conservation of Resources (COR) theory provides the central lens through which this study interprets teacher-AI collaboration and its consequences. COR maintains that people strive to obtain, retain, and protect resources—broadly defined as objects, personal characteristics, conditions, or energies that they value in themselves or as means to other valued ends (Hobfoll & Shirom, 2000). When teachers experience a net gain in resources—extra time, reduced cognitive load, enhanced professional competence—COR predicts a “resource-gain spiral” that generates positive affect, motivation, and performance. Conversely, when resource demands exceed returns, a “resource-loss spiral” sets in, producing stress and eventual disengagement. Because gains and losses rarely occur in isolation, COR also emphasizes resource caravans—clusters of interrelated resources that travel together—and resource caravan passageways, namely, organizational contexts that facilitate or impede resource exchange.

Within this framework, teacher-AI collaboration represents a pivotal object/conditional resource. Intelligent systems can automate routine grading and analytics, potentially freeing teachers’ time and energy for creative planning and individualized feedback; these savings constitute the first link in a possible gain spiral. Yet the same collaboration can impose hidden costs: mastering new software, monitoring algorithmic outputs, and troubleshooting technical glitches may lengthen preparation time and threaten instructional autonomy, thereby initiating a loss spiral. Whether collaboration ultimately enriches or depletes a teacher’s resource reservoir therefore depends on complementary personal and contextual factors.

The model proposed here positions technology self-efficacy as a key personal resource that converts collaboration into engagement. Teachers confident in their ability to harness AI are more likely to translate time savings into renewed instructional investment, whereas those with low self-efficacy may experience identical tools as resource drains. At the contextual level, perceived organizational support operates as a resource caravan passageway: generous training, technical assistance, and emotional backing lower the entry costs of AI adoption and magnify its benefits; inadequate support leaves teachers to shoulder those costs alone. Finally, teaching engagement is conceptualized as the resource-investment outcome that closes the spiral: only when teachers judge their resource balance to be favorable will they willingly reinvest cognitive, emotional, and behavioral energy in classroom activities.

By embedding collaboration (object resource), self-efficacy (personal resource), organizational support (contextual passageway), and engagement (investment outcome) within a single COR logic, the study explains the entire moderated-mediation structure. A direct collaboration → engagement path captures the net balance of gains and losses; an indirect collaboration → self-efficacy → engagement path reflects resource conversion; and the moderation of collaboration by organizational support illustrates how passageways shape resource caravans. Thus, COR not only unifies the model’s constructs but also clarifies why teacher-AI collaboration can be a double-edged sword whose final impact on teaching engagement hinges on the dynamic interplay of gains, losses, and supportive conditions.

### 2.2. Teacher-AI Collaboration and Teaching Engagement

Currently, collaboration between teachers and artificial intelligence (AI) has become an important approach to improving teaching efficiency and quality (Zawacki-Richter et al., 2019). Teacher-AI collaboration is reflected not only in technical cooperation—such as data analysis and assignment grading assistance—but also in the restructuring of teaching philosophies and professional roles (Lee et al., 2024). Through effective human–machine collaboration, AI can help teachers manage repetitive tasks and provide personalized instructional recommendations, thereby saving cognitive and time resources and allowing teachers to focus on more creative and interactive teaching activities (Pisica et al., 2023).

Based on the Conservation of Resources (COR) Theory, individuals tend to acquire and utilize resources to protect existing ones and enhance adaptability (Hobfoll & Shirom, 2000). In the educational context, collaboration with AI provides teachers with an “alternative external resource” that reduces resource consumption (e.g., energy, emotional stress) during task execution and stimulates active engagement in teaching activities (Fowler, 2023). For instance, with the assistance of AI, teachers can quickly obtain student learning data, enabling more efficient instructional diagnosis and feedback, which enhances their sense of control and professional value in teaching.

Moreover, AI collaboration can generate cognitive motivation. When working with AI, teachers may experience improved efficiency and visible outcomes, which enhance their sense of achievement and professional identity. These positive experiences further reinforce their teaching engagement (Borg, 2010). Teaching engagement refers to the degree of attention, enthusiasm, and sustained investment that teachers demonstrate in their teaching (Kangas et al., 2017). Prior studies suggest that when external supportive resources are sufficient, individuals are more likely to exhibit high levels of work engagement and positive behaviors (Hobfoll & Shirom, 2000). Therefore, teacher-AI collaboration can relieve pressure on teaching resources, optimize instructional processes, and enhance positive emotions and professional identity, thereby promoting higher levels of teaching participation.

**Hypothesis 1** **(H1).** *Teacher-AI collaboration positively predicts teachers’ teaching engagement.*

### 2.3. The Mediating Role of Technological Self-Efficacy

According to the Conservation of Resources Theory, when individuals face environmental change or technological challenges, they engage in learning and adaptation behaviors to acquire new resources and improve their coping capacity (Hobfoll & Shirom, 2000). Teacher-AI collaboration is essentially a resource acquisition process in practice. Through hands-on experience with AI tools and participation in AI-assisted instructional design, teachers can develop a stronger sense of control over technology and gradually form the belief that “I can successfully use AI in teaching” (Fokkens-Bruinsma & Canrinus, 2014). This belief, known as technological self-efficacy (Alharbi & Drew, 2019), reflects an individual’s confidence in handling AI-related teaching tasks. In other words, positive interactions and collaborative experiences with AI help teachers form a stable and positive sense of self-efficacy, which serves as a key psychological resource (Yalcin et al., 2011).

Technological self-efficacy is not only a cognitive judgment but also a resource-based psychological state that influences teachers’ attitudes and behavioral responses toward technology integration in teaching (Gökçek et al., 2013). According to COR Theory, when individuals possess sufficient internal resources (such as efficacy beliefs), they are more likely to adopt proactive coping strategies when facing challenges, leading to higher levels of investment and sustained behavior (Hobfoll & Shirom, 2000). Teachers with high technological self-efficacy tend to explore the potential of AI in instruction, enhancing their sense of control, goal orientation, and achievement in the teaching process, thereby stimulating emotional engagement and enthusiasm for teaching (Cherry & Flora, 2017). Conversely, a lack of efficacy may lead to technology-related anxiety, avoidance behaviors, and reduced teaching engagement (Gomez et al., 2022).

Therefore, technological self-efficacy acts as a crucial individual resource that can significantly enhance teachers’ motivation and willingness to participate in teaching under the AI-integrated environment. According to COR Theory, there exists a transmission path among different types of resources: external resources (such as opportunities for AI collaboration) can influence behavioral outcomes (such as teaching engagement) through internal psychological resources (such as self-efficacy) (Hobfoll & Shirom, 2000). The confidence and perceived competence developed through teacher-AI collaboration translate into a psychological driving force that enhances teaching involvement—marking the resource transformation from “tool use” to “active teaching” (Huang et al., 2020).

**Hypothesis 2** **(H2).** *Technological self-efficacy mediates the relationship between teacher-AI collaboration and teaching engagement.*

### 2.4. The Moderating Role of Perceived Organizational Support

While teacher-AI collaboration has become increasingly prevalent, for many teachers, it represents not just a change in work methods but a fundamental challenge to their existing knowledge systems and instructional beliefs (Xu et al., 2024). As AI systems intervene in multiple instructional processes such as lesson planning, classroom interaction, and assessment, teachers face pressure to learn new technologies, reconstruct their professional identity, and manage fluctuations in their sense of control over instruction (Tatar et al., 2009). According to COR Theory, whether individuals can adapt and grow in the face of environmental stress depends on their ability to mobilize and convert resources (Hobfoll & Shirom, 2000). Although AI collaboration may offer efficiency gains and teaching innovation, if corresponding support is lacking, teachers may perceive it as a resource threat, leading to anxiety, self-doubt, and resistance—thus inhibiting the formation of technological self-efficacy (Pan & Chen, 2021).

Against this backdrop, perceived organizational support (POS) becomes a critical external resource for teachers coping with technological transitions. POS includes not only “functional support” (e.g., policies, training, technical infrastructure) but also “emotional support” (e.g., encouragement, trust, and recognition) (Nayir, 2012). COR Theory emphasizes that external resources can stimulate the generation of internal resources. When teachers perceive a supportive and tech-friendly organizational environment—with ample training, technical assistance, and tolerance for experimentation—they are more likely to view AI collaboration as a “controllable opportunity” rather than an “uncontrollable threat” (Demir, 2015). In such safe and supportive environments, teachers are more willing to explore technologies, accept failures, and grow through reflection. These experiences gradually build a positive perception of their technological abilities—i.e., technological self-efficacy (Farooqi et al., 2019).

For instance, schools that provide AI teaching demonstrations, hold technology exchange workshops, offer real-time technical assistance, and establish feedback mechanisms can significantly reduce teachers’ sense of helplessness and frustration, thereby enhancing their perceived control and self-efficacy in using AI tools.

Thus, as a form of social resource, perceived organizational support not only directly affects teachers’ attitudes and behaviors toward AI collaboration but also plays a moderating role in the relationship between teacher-AI collaboration and technological self-efficacy (Keskinkılıç Kara et al., 2015). When POS is high, teachers are more likely to have positive experiences from AI collaboration and convert them into confidence and perceived competence, resulting in higher technological self-efficacy (Holsblat, 2014). In contrast, under low-POS conditions, even with opportunities for AI collaboration, teachers may lack sufficient support or emotional security to transform their experiences into increased efficacy—thus weakening this relationship (Bibi et al., 2019).

**Hypothesis 3** **(H3).** *Perceived organizational support positively moderates the effect of teacher-AI collaboration on technological self-efficacy. Specifically, the positive relationship is stronger when perceived organizational support is high and weaker when it is low.*

As previously discussed, teacher-AI collaboration enhances teaching engagement through increased technological self-efficacy, forming a stable mediation mechanism. However, according to COR Theory, the resource acquisition process is not linear but influenced and amplified by other resource levels (Hobfoll & Shirom, 2000). Specifically, whether individuals can effectively convert collaborative experiences into efficacy beliefs depends significantly on the availability of external support resources. In this context, perceived organizational support acts as a critical moderating resource (Anomneze et al., 2016). When teachers perceive a high level of organizational support—including training systems, technical resources, managerial encouragement, and a supportive culture—they are more likely to gain positive experiences from AI collaboration and internalize them into the belief that “I am capable of teaching with AI” (Pradesa et al., 2023). This self-efficacy, in turn, fuels their enthusiasm, sense of responsibility, and sustained investment in teaching—i.e., teaching engagement (Zhang et al., 2023).

Therefore, perceived organizational support not only moderates the direct relationship between teacher-AI collaboration and technological self-efficacy but also moderates the indirect effect of teacher-AI collaboration on teaching engagement through technological self-efficacy. In contrast, in low-POS environments, teachers may fail to derive self-efficacy from AI collaboration—or even perceive it as an additional burden—thus weakening or disrupting the positive psychological effects of collaboration and reducing their impact on teaching engagement.

**Hypothesis 4** **(H4).** *Perceived organizational support moderates the indirect effect of teacher-AI collaboration on teaching engagement via technological self-efficacy. This indirect effect is stronger under high levels of perceived organizational support and weaker under low support conditions.*

**Figure 1:**
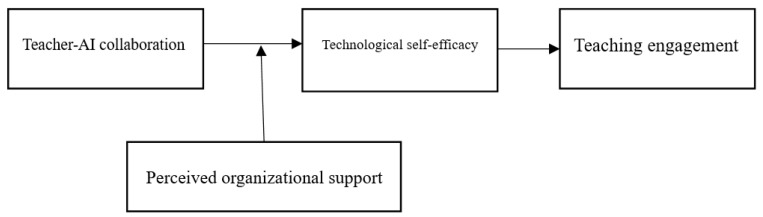
Research model.

Accordingly, the conceptual model of this study is illustrated in Figure 1.

## 3. Method

### 3.1. Sample and Procedure

This study employed a questionnaire survey to explore the relationships among teacher-AI collaboration, technological self-efficacy, perceived organizational support, and teaching engagement. Data collection was conducted from October to December 2024 across several universities in Zhejiang Province. Specifically, six institutions were invited: three comprehensive research universities, two provincial teaching-oriented universities, and one privately funded applied university. All six are participants in the province’s “Smart Education 2.0” initiative, which mandates the integration of AI into at least 20 percent of undergraduate courses by 2025. To ensure disciplinary breadth, we used stratified sampling that covered 12 academic fields, including engineering, computer science, business, foreign languages, education, medicine, and the arts. Each department provided a roster of full-time instructors who had used AI tools in at least one course for a full semester; from these rosters we randomly selected respondents, yielding 536 valid questionnaires. The study targeted institutions that had implemented AI-assisted teaching technologies, including, but not limited to, intelligent education platforms, AI-assisted grading systems, and virtual classrooms. Typical tools in use were adaptive learning systems for foundational mathematics, automated essay-scoring platforms for language courses, speech-recognition modules for oral-proficiency assessment, and VR/AR simulation labs for engineering design. All participating universities required faculty to complete a 16 h training program on these tools and provided centralized technical support labs to troubleshoot classroom issues. Consequently, each respondent had experienced at least one full academic year of AI deployment, allowing for a realistic assessment of both the benefits and the challenges associated with teacher-AI collaboration. These universities were actively engaged in digital transformation initiatives aimed at improving teaching quality and efficiency.

A stratified random sampling method was adopted to ensure representation across various disciplines such as the humanities, sciences, engineering, and business. Questionnaires were randomly distributed to frontline faculty members, with the target population being university instructors—particularly those involved in or utilizing AI tools in their teaching practices. In total, 600 questionnaires were distributed across six undergraduate institutions located in Zhejiang Province. Over a two-month data collection period, 536 valid responses were received, yielding a valid response rate of 89.3%.

Demographic information of the valid sample is as follows: The valid sample comprised 536 teachers, of whom 311 (58.0%) were female and 225 (42.0%) were male. The age distribution was relatively balanced, with 52.3% of participants aged between 30 and 45, and 31.4% aged 46 or older; the remaining respondents were younger than 30 years old. In terms of teaching experience, 49.5% had more than 10 years of teaching experience, indicating that the sample had substantial instructional background. Regarding disciplinary distribution, 36.7% were from the humanities, 43.5% from science and engineering, and 19.8% from business-related fields.

### 3.2. Measures

This study utilized standardized scales to measure teacher-AI collaboration, technological self-efficacy, perceived organizational support, and teaching engagement. All instruments were adapted from existing literature and refined through preliminary investigations. A 5-point Likert scale was used for all items (1 = Strongly Disagree, 5 = Strongly Agree). All items are shown in Appendix A. Details of each scale are provided below:

Teacher-AI Collaboration was assessed using a scale adapted from Kong et al. (2023). A sample item is: “AI participates in my decision-making process.” The reliability of this scale was high, with Cronbach’s α = 0.88, indicating good internal consistency.

Technological Self-Efficacy was measured using a scale developed by Laver et al. (2012), which evaluates teachers’ confidence in, and perceived ability to use, AI technologies. Sample items include: “I believe I can independently use AI tools for classroom teaching” and “I feel confident in solving technical problems encountered when using AI.” The scale demonstrated excellent reliability (α = 0.92).

Perceived Organizational Support was assessed by adapting the scale from Shore and Tetrick (1991) to the educational context. This scale measures the support perceived by teachers from their institutions during technological transformation, encompassing emotional support, training opportunities, and resource provision. Sample items include: “My university values my contribution when I use AI in teaching,” and “Management encourages and supports us in using new technologies in the classroom.” The scale showed high internal reliability (α = 0.90).

Teaching Engagement was measured using a scale adapted from Kelly et al. (2022), revised to fit the context of AI-assisted teaching. This construct includes emotional, cognitive, and behavioral dimensions of engagement. Sample items are: “I feel very excited and engaged when using AI in my teaching,” and “Using AI makes me enthusiastic about lesson planning.” The reliability coefficient was α = 0.91, indicating strong internal consistency.

To account for potential confounding effects on the relationships among teacher-AI collaboration, technological self-efficacy, perceived organizational support, and teaching engagement, this study included several control variables: age, gender, educational background, and years of teaching experience. These variables are commonly used in empirical studies in the field of education and are effective in controlling for the influence of individual background characteristics on teaching behaviors and attitudes.

## 4. Results

### 4.1. Common Method Bias Test

To assess potential common method bias, we first conducted Harman’s single-factor test. All measurement items were subjected to an unrotated exploratory factor analysis. The results showed that the first principal component had an eigenvalue greater than 1 and explained 16.63% of the total variance, which is well below the critical threshold of 40%. This suggests that common method bias is not a serious concern in this study.

To further verify this finding, given the potential insensitivity of Harman’s test, we introduced a method factor into the five-factor model and compared it with the original five-factor model. The comparison revealed minimal differences in fit indices (ΔCFI = 0.021, ΔTLI = 0.019, ΔRMSEA = 0.009), providing additional evidence that common method bias is not a significant issue in this study.

### 4.2. Descriptive Statistics

Table 1 presents the means, standard deviations, and correlation coefficients for all variables included in the study. As shown, Teacher-AI collaboration is significantly and positively correlated with Technological Self-Efficacy (r = 0.32, *p* < 0.01), Teaching Engagement (r = 0.31, *p* < 0.01), and Perceived Organizational Support (r = 0.30, *p* < 0.01). In addition, Technological Self-Efficacy is significantly positively correlated with Teaching Engagement (r = 0.37, *p* < 0.001). These results provide preliminary support for the study’s hypotheses.

For all latent study variables—Teacher-AI Collaboration, Technological Self-Efficacy, Perceived Organizational Support, and Teaching Engagement—each respondent’s score was first calculated as the arithmetic mean of the corresponding 5-point Likert items (1 = Strongly Disagree to 5 = Strongly Agree) listed in Appendix A; the values shown in Table 1 are the grand means and standard deviations of these composite scores across the full sample (N = 536).

### 4.3. Confirmatory Factor Analysis

Confirmatory factor analysis (CFA) was conducted using Mplus 7.4. A four-factor model (the baseline model) was first constructed, followed by comparisons with alternative models, including a three-factor model, a two-factor model, and a one-factor model. The results are presented in Table 2.

Among the tested models, the four-factor model demonstrated significantly better fit indices than the alternative models (χ^2^/*df* = 1.11, RMSEA = 0.04, CFI = 0.96, TLI = 0.95), indicating good discriminant validity among the main variables. These results confirm that the measurement model fits the data well and that the variables are empirically distinct.

### 4.4. Hypothesis Testing

**Figure 2:**
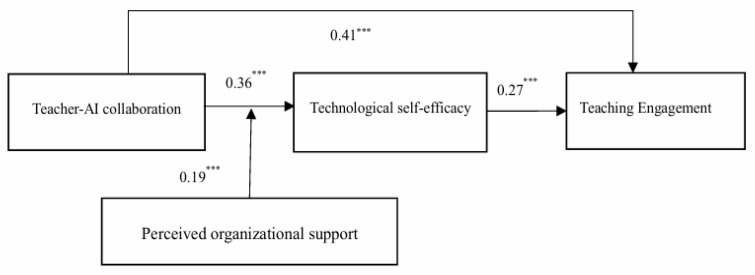
The result of hypothesis test. (Notes: *** *p* < 0.001).

This study employed Mplus 7.4 to construct a structural equation model for hypothesis testing. The path coefficients and their significance levels are shown in Figure 2.

#### 4.4.1. Direct Effects Testing

As shown in Figure 2, Teacher-AI collaboration has a significant positive impact on Technological Self-Efficacy (B = 0.36, *p* < 0.001) and Teaching Engagement (B = 0.41, *p* < 0.001). A one-unit increase on the 1–5 collaboration scale corresponds to roughly a 0.70-SD rise in self-efficacy and a 0.78-SD rise in engagement, evidence of a practically meaningful boost to teachers’ confidence and classroom vitality. Interpreted through COR theory, collaboration operates as a resource-gain trigger: time freed by AI and the experience of successful human–machine teamwork supply fresh competence resources, which teachers then reinvest in their instructional practice. Therefore, H1 is supported. In addition, Technological Self-Efficacy significantly and positively affects Teaching Engagement (B = 0.27, *p* < 0.001). This coefficient translates to an average 0.5-SD increase in engagement for every unit of self-efficacy gained, underscoring that teachers’ belief in their ability to handle AI is a pivotal conduit for turning technological affordances into observable teaching behaviors.

#### 4.4.2. Mediating Effect Testing

To verify the mediating role of Technological Self-Efficacy in the relationship between Teacher-AI collaboration and Teaching Engagement, this study applied the conditional indirect effects method recommended by Preacher (Bootstrap = 5000). The results showed that the mediating effect of Technological Self-Efficacy between Teacher-AI collaboration and Teaching Engagement was 0.42, with a 95% confidence interval of [0.29, 0.57], which does not include 0. The indirect path accounts for about 51% of the total collaboration → engagement link, implying that more than half of the motivational lift generated by AI teamwork travels through teachers’ strengthened technological confidence rather than through a direct workload reduction alone. This indicates a significant mediating effect of Technological Self-Efficacy. Therefore, H2 is supported.

#### 4.4.3. Moderating Effect Testing

As shown in Figure 2, Perceived Organizational Support moderates the relationship between Teacher-AI collaboration and Teaching Engagement (interaction term coefficient B = 0.19, *p* < 0.001). Simple-slope analysis indicates that the positive effect of collaboration on engagement is nearly twice as steep (+0.55) when organizational support is one SD above the mean, compared with low-support environments (+0.29). This pattern suggests that a supportive climate functions as a “resource-caravan passageway”, amplifying the gains teachers extract from AI collaboration. Therefore, H3 is supported.

#### 4.4.4. Moderated Mediation Effect Testing

This study used the Bootstrap method with 5000 random samples to test the moderated mediation effect. As shown in Table 3, when Perceived Organizational Support is low, the mediating effect of Technological Self-Efficacy is 0.22, with a 95% confidence interval of [0.12, 0.39], excluding 0; when Perceived Organizational Support is high, the mediating effect is 0.12, with a 95% confidence interval of [−0.14, 0.11], including 0. Taken together with the significant interaction reported above, these results indicate that high organizational support channels a portion of the collaboration benefit directly into engagement, leaving a smaller share to flow indirectly through self-efficacy, whereas low support makes teachers’ personal sense of competence the critical hinge for converting collaboration into engagement. The difference between the two is significant. Therefore, H4 is supported.

## 5. Discussion

Our findings show that teacher-AI collaboration (TAC) exerts a direct and positive influence on teaching engagement (TE); teachers who work more closely with AI report higher cognitive, emotional, and behavioral investment in their classes. This echoes evidence that AI tools can augment instructional effectiveness (Aler Tubella et al., 2024; Kong et al., 2023) while extending the conversation to teachers’ own work attitudes, which earlier studies often overlooked (Crompton & Burke, 2023; Fowler, 2023). Viewed from a broader behavioral-science lens, this pattern parallels findings in other knowledge-intensive domains—such as finance analysts partnering with predictive algorithms or clinicians consulting diagnostic engines—where human–machine teaming acts as a situational resource that employees reinvest in performance-relevant behaviors. In line with Conservation of Resources logic, TAC therefore functions as an external resource that educators—and, by extension, many professional workers—reinvest in day-to-day practice, thereby raising engagement.

The analysis further establishes technological self-efficacy (TSE) as a partial mediator: collaboration with AI heightens teachers’ confidence in managing digital tools, and this heightened confidence, in turn, boosts engagement. Beyond the school setting, the same psychological mechanism is pertinent to any workplace where algorithmic systems are being introduced; self-efficacy theory predicts—and our data confirm—that perceived capability is the channel through which collaboration with intelligent agents translates into discretionary effort (Anomneze et al., 2016; Bates et al., 2020; Gökçek et al., 2013; Laver et al., 2012). Thus, the present study offers a transferable account of how AI exposure reshapes workers’ beliefs and amplifies their task-focused energy, informing behavioral-science debates on technology-driven motivation.

Finally, perceived organizational support (POS) strengthens the link between TAC and TSE, confirming that institutional training, resources, and leadership encouragement amplify the confidence that users derive from AI (Nayir, 2012; Zhang et al., 2023). When support is high, the positive slope from TAC to TSE nearly doubles; when support is low, the slope flattens, and the indirect TAC → TSE → TE pathway weakens. This pattern reinforces the COR notion that supportive contexts act as resource-caravan passageways (Hobfoll & Shirom, 2000), enabling employees in any sector to convert technological collaboration into personal efficacy and, ultimately, higher engagement. Without such passageways, workers may still gain from AI, but the returns are smaller and more fragile—an insight especially salient in rapidly digitalizing environments such as those now prevalent in China (Xu et al., 2024; Farooqi et al., 2019). Hence, our education-based results generalize to behavioral-science questions about how organizational climates condition the motivational pay-off from human–AI co-intelligence in diverse professional settings.

### 5.1. Theoretical Contributions

First, this study weaves Conservation of Resources (COR) theory into the fast-evolving literature on educational AI, demonstrating that teacher-AI collaboration (TAC) is simultaneously a material resource—removing repetitive grading and scheduling demands—and a psychological resource generator that boosts teachers’ technological self-efficacy. By showing that these dual gains launch a resource-gain spiral culminating in higher behavioral engagement, we move COR beyond its traditional workplace applications and situate it firmly within digital-era instructional work, where resources are co-created by humans and algorithms rather than transferred only between people (Ma et al., 2024; Hobfoll & Shirom, 2000).

Second, the work reorients the ed-tech conversation away from system performance metrics toward educator-centered processes. Prior AI studies have largely celebrated improved student scores or algorithmic accuracy (Aler Tubella et al., 2024; Kong et al., 2023); our model instead spotlights the teacher, detailing how day-to-day collaboration with AI reshapes professional motivation, effort allocation, and classroom routines. This shift directly answers recent calls to illuminate the human behavioral changes that accompany technological transformation, rather than treating teachers as passive adopters (Crompton & Burke, 2023; Fowler, 2023).

Third, by establishing technological self-efficacy (TSE) as the psychological bridge between TAC and engagement, the study enriches self-efficacy theory in educational psychology. Our evidence clarifies that AI use is not merely a skills upgrade; it fortifies teachers’ beliefs in their own competence, energizing broader professional activities and experimentation (Gökçek et al., 2013; Laver et al., 2012). This mechanism is especially salient amid China’s policy-driven push toward digital classrooms, where heightened TSE can offset the anxiety and resistance often triggered by rapid technological mandates (Xu et al., 2024).

### 5.2. Practical Implications

First, the study finds that teacher-AI collaboration significantly enhances teaching engagement, offering clear direction for educational administrators. Amid rapid digital reform, many teachers still struggle with effectively adopting AI or only engage with it on a superficial level. These findings suggest that schools should systematically promote AI-assisted teaching models, such as integrating AI in lesson planning, resource organization, and personalized student support. By establishing human–machine collaborative mechanisms, schools can stimulate teachers’ enthusiasm and proactivity. Administrators should also use case sharing and model classrooms to demonstrate AI’s effectiveness in reducing workload and improving efficiency, thereby enhancing willingness to collaborate and motivation for teaching. These recommendations are applicable across both K-12 and higher education contexts, especially for implementing AI platforms and localized tools (e.g., personalized assessment systems or teaching assistants).

Second, the identified mediating role of technological self-efficacy highlights that increasing teachers’ understanding of, and confidence in, using technology is critical to improving their teaching engagement. This is particularly relevant where teachers lack technical backgrounds or remain skeptical about AI. Schools should establish multi-level, staged training systems that help teachers transition from basic to proficient users—for instance, offering targeted AI training, micro-course design workshops, or appointing “instructional technology mentors” for ongoing personalized support. Incentive systems, such as performance-based recognition or resource allocation for technologically adept teachers, can further strengthen positive perceptions and confidence in tech use. By cultivating a sense that “technology is learnable and controllable”, teachers are more likely to embrace emerging tools and integrate them into their instruction, naturally boosting engagement.

Lastly, the study shows that perceived organizational support significantly moderates the relationship between teacher-AI collaboration and technological self-efficacy, indicating that a supportive environment is a crucial external resource for teachers adapting to change. The introduction of AI essentially reallocates teachers’ resource systems and may increase cognitive and emotional burdens. Based on Conservation of Resources Theory, this study suggests that schools should foster a resource-enriched organizational climate by focusing on institutional design, leadership style, and humanistic care. Specifically, schools should ensure clear and stable technology-use policies and reliable AI systems; leaders should express understanding and patience toward teachers’ adaptation processes, offering both emotional and material support; psychological counseling services and peer-support groups can help alleviate stress and isolation during transitions. This is especially vital in rural or under-resourced areas, where organizational support can greatly ease the burden of adopting new technologies and sustain teacher confidence and engagement.

### 5.3. Limitations and Future Research Directions

This study adopts a cross-sectional survey design, which, while validating the impact mechanism of teacher-AI collaboration on teaching engagement, cannot fully capture the causal and dynamic nature of these relationships over time. In practice, teaching engagement and self-efficacy may be influenced by long-term adaptation, feedback, and experience accumulation. Future research could use longitudinal designs to explore the long-term pathways of teacher-AI collaboration, or adopt experimental or intervention methods to enhance causal inference and uncover dynamic mechanisms more accurately.

While this study focuses on the mediating effect of technological self-efficacy and the moderating effect of perceived organizational support, it only reveals part of the psychological and organizational mechanisms involved. Teachers’ professional identity, learning motivation, and innovation consciousness may also play important roles in mediating the relationship between AI use and teaching behavior. Similarly, other organizational factors—such as infrastructure quality, digital culture, or leadership style—could further influence the effectiveness of AI-supported teaching. Future studies could employ multilevel models (e.g., MLM) to explore individual–organizational interaction mechanisms and expand theoretical boundaries, offering more comprehensive insights into educational technology integration.

Additionally, this study primarily draws on data from teachers in eastern China, where educational resources, technical infrastructure, and policy support are relatively abundant. Therefore, the findings may not fully reflect the realities and challenges faced by teachers in less developed or rural areas. Differences in educational level (e.g., higher education or vocational education) may also affect the way AI is used and perceived, given differences in teaching goals, assessment standards, and student populations. Future research could conduct comparative studies across regions and education levels to identify context-specific strategies and boundary conditions for effective AI integration in teaching.

## 6. Conclusions

With the accelerated integration of artificial intelligence into educational practice, teacher-AI collaboration has become an important driver of educational modernization and a natural laboratory for behavioral-science research on human adaptation to intelligent agents. However, empirical research on teachers’ behavioral and psychological responses in AI-enabled environments remains limited. Based on the Conservation of Resources Theory, this study proposes and verifies a theoretical model incorporating a mediating variable (technological self-efficacy) and a moderating variable (perceived organizational support) to examine how teacher-AI collaboration influences teaching engagement, as well as the underlying mechanisms and boundary conditions that govern resource-based behavior change.

The results show that teacher-AI collaboration significantly predicts teaching engagement, indicating that AI is not merely a tool but a situational resource that reshapes effort allocation, emotional investment, and classroom behavior. This highlights the positive role of human–machine collaboration in motivating teaching behavior and responds to the urgent need to redefine the teacher’s role in the digital age. Further analysis reveals that technological self-efficacy mediates the relationship between collaboration and engagement, suggesting that working with AI not only improves technical competence but also enhances teachers’ confidence, which, in turn, energizes proactive teaching behaviors such as experimentation, reflection, and student mentoring. This demonstrates how external technological resources are internalized into psychological resources and subsequently expressed in observable behavior, providing new insights into how AI empowers teaching.

Additionally, perceived organizational support significantly moderates the collaboration-to-self-efficacy link. When organizational support is strong, teachers are more likely to internalize collaborative experiences into self-efficacy, echoing behavioral–science evidence that supportive climates amplify the motivational impact of new technology by widening “resource-caravan passageways”. The moderated-mediation effect confirms that perceived support not only directly enhances technological confidence but also indirectly shapes the teaching engagement by facilitating the conversion of external resources into sustained behavioral investment.

In conclusion, this study deepens our understanding of the behavioral pathways through which teacher-AI collaboration affects teaching engagement and provides both theoretical insights and practical guidance for optimizing AI-supported work environments more broadly. Future development of educational technology should therefore prioritize teachers’ psychological perceptions and resource access, fostering a triadic “technology–teacher–organization” ecosystem in which behavioral engagement, not mere adoption, becomes the central marker of successful digital transformation.

## Figures and Tables

**Table 1:** Means, standard deviations, correlations, and reliability among study variables.

Variable	Mean	SD	1	2	3	4	5
1. Age	28.34	2.23	1				
2. Teacher-AI collaboration	4.01	1.22	0.11	1			
3. Teaching engagement	4.07	1.02	0.21	0.31 **	1		
4. Technological self-efficacy	4.30	0.42	0.13 *	0.32 **	0.37 ***	1	
5. Perceived organizational support	3.23	1.23	0.21	0.30 **	0.40 **	0.32 **	1

Notes: * *p* < 0.05; ** *p* < 0.01; *** *p* < 0.001.

**Table 2:** Confirmatory factor analysis.

Model	χ^2^/df	CFI	TLI	RMSEA
Four-factor model	1.11	0.96	0.95	0.04
Three-factor model	10.11	0.80	0.75	0.13
Two-factor model	14.57	0.53	0.53	0.21
One-factor model	18.70	0.47	0.41	0.34

**Table 3:** Moderated mediation effect test results.

Moderator	Effect	SE	Lower Limit of 95% Confidence Interval	Higher Limit of 95% Confidence Interval
Mean − 1SD	0.22	0.04	0.12	0.39
Mean + 1SD	0.12	0.49	−0.14	0.11

## Data Availability

The data supporting the findings of this study can be obtained by contacting the first author.

## Appendix A

**Items of Teacher-AI Collaboration**

1. AI participates in my decision-making process.
2. AI participates in my prediction process.
3. AI participates in my problem-solving process.
4. AI participates in my information identification and evaluation process.
5. AI participates in my problems, opportunities, or risk recognition process.

**Items of Teaching Engagement**

1. I feel very excited and engaged when using AI in my teaching.
2. Using AI makes me enthusiastic about lesson planning.
3. AI-assisted teaching makes me feel professionally fulfilled.
4. I actively think about how AI can improve my instructional methods.
5. I regularly reflect on my teaching practice to better use AI tools.
6. I set challenging goals for myself when integrating AI into teaching.
7. I actively explore AI tools to improve my instructional methods.
8. I spend extra time outside class to learn about AI technologies.
9. I share AI-teaching experiences with colleagues.

**Items of Technological Self-Efficacy**

1. I believe I can independently use AI tools for classroom teaching.
2. I feel confident in solving technical problems encountered when using AI.
3. I am able to learn new AI functions quickly.
4. I can effectively integrate AI functions into my lesson plans.
5. I can adjust AI system settings to meet different teaching needs.
6. I can guide students in using AI tools safely and effectively.

**Items of Perceived Organizational Support**

1. My university values my contribution when I use AI in teaching.
2. The university provides sufficient training resources to support the use of AI technologies.
3. Management encourages and supports us in using new technologies in the classroom.
4. Technical staff respond promptly when I encounter AI-related problems.
5. My university is willing to allocate funds to improve AI tools for teaching.
6. The leadership shows concern for my well-being when implementing new AI systems.
